# The characteristics of the HIV subtype B epidemic in Slovenia

**DOI:** 10.1186/1471-2334-14-S4-O23

**Published:** 2014-05-29

**Authors:** Maja M Lunar, Ana B Abecasis, Anne-Mieke Vandamme, Janez Tomažič, Ludvik Vidmar, Primož Karner, Tomaž D Vovko, Blaž Pečavar, Mario Poljak

**Affiliations:** 1 Institute of Microbiology and Immunology, Faculty of Medicine, University of Ljubljana, Ljubljana, Slovenia; 2 Instituto de Higiene e Medicina Tropical, Centro de Malária e Outras Doenças Tropicais, Universidade Nova de Lisboa, Lisbon, Portugal; 3 Clinical and Epidemiological Virology, Rega Institute for Medical Research, K. U. Leuven, Leuven, Belgium; 4 Department of Infectious Diseases, University Medical Center Ljubljana, Ljubljana, Slovenia

## 

Slovenia is a small Central European country with a relatively modest burden of HIV disease, with fewer than 1 per 1,000 inhabitants infected. The HIV epidemic mostly affects men who have sex with men (MSM), with subtype B as the most represented subtype in over 85% of patients. The aim of this study was to establish the properties of the subtype B HIV epidemic in Slovenia up to the end of 2012.

For the purpose of this study, data and sequences were gathered from 3 previous studies conducted in Slovenia examining the prevalence of transmitted drug resistance among therapy naïve HIV-1 positive patients diagnosed in the years 2000-2012. Only subtype B sequences were selected for this study (determined by the REGA HIV-1 Subtyping tool, v2.0), a total of 223 partial pol gene sequences were included, representing 52% of all patients newly diagnosed in 13 years.

The maximum likelihood (ML) phylogenetic tree was constructed using PhyML 3.0 and transmission clusters were identified according to the approximate likelihood ratio test branch support values (>0.90). The Monte Carlo Markov chain method available in the BEAST package v1.7.1 was employed, using a relaxed clock model with uncorrelated lognormal distribution and the Bayesian skyline coalescent model. The clusters previously identified in the ML analysis were reviewed and confirmed according to posterior probability values (>0.990).

Combined analysis (ML and Bayesian analysis) revealed 8 major clusters (n≥10 patients), 1 group of 4 patients, 2 trios and 12 transmission pairs. Among 223 included individuals, 146 (65.5%) patients belonged to large transmission clusters comprising 10 or more individuals and 34 (15.2%) patients to small clusters of 2-4 patients, leaving only 43 (19.3%) of the Slovenian patients infected with subtype B without an epidemiological link observed by phylogenetic inference. Statistical analysis examining the characteristics of patients found in large clusters revealed significantly fewer patients in a cluster diagnosed prior to 2005 (p=0.0388) and significantly more patients reported Slovenia as the country in which the infection occurred (p<0.0001) compared to other countries.

In conclusion, several introductions of HIV subtype B occurred in Slovenia. The majority of patients were found with a transmission link, exhibiting a closed HIV community, with the virus being transmitted predominantly between individuals within Slovenia.

